# Species-Specific Induction of Plant Volatiles by Two Aphid Species in Apple: Real Time Measurement of Plant Emission and Attraction of Lacewings in the Wind Tunnel

**DOI:** 10.1007/s10886-021-01288-5

**Published:** 2021-07-01

**Authors:** Zaid Badra, Sebastian Larsson Herrera, Luca Cappellin, Franco Biasioli, Teun Dekker, Sergio Angeli, Marco Tasin

**Affiliations:** 1 Faculty of Science and Technology, Free University of Bozen-Bolzano, Piazza Università 1, 39100 Bolzano, Italy; 2 Dep. of Plant Protection Biology, Swedish University of Agricultural Science, Box 102, 230 53 Alnarp, Sweden; 3 Research and Innovation Centre, Fondazione Edmund Mach (FEM), Via E. Mach 1, 38010 San Michele All’Adige, Italy; 4 Department of Chemistry, University of Padova, Via Marzolo 1, 35131 Padova, Italy

**Keywords:** Acetic acid, *Aphis pomi*, *Chrysoperla carnea*, *Dysaphis plantaginea*, Proton-Transfer-Reaction—Time of Flight—Mass Spectrometry, 2-phenylethanol, DMNT, Terpenoids, Wind tunnel

## Abstract

Upon damage by herbivores, plants release herbivory-induced plant volatiles (HIPVs). To find their prey, the pest’s natural enemies need to be fine-tuned to the composition of these volatiles. Whereas standard methods can be used in the identification and quantitation of HIPVs, more recently introduced techniques such as PTR-ToF–MS provide temporal patterns of the volatile release and detect additional compounds. In this study, we compared the volatile profile of apple trees infested with two aphid species, the green apple aphid *Aphis pomi*, and the rosy apple aphid *Dysaphis plantaginea*, by CLSA-GC–MS complemented by PTR-ToF–MS. Compounds commonly released in conjunction with both species include nonanal, decanal, methyl salicylate, geranyl acetone, (*Z*)-3-hexenyl acetate, (*Z*)-3-hexenyl butanoate, (*Z*)-3-hexenyl 2-methyl-butanoate, (*E*)-β-caryophyllene, β-bourbonene and (*Z*)-3-hexenyl benzoate. In addition, benzaldehyde and (*E*)-β-farnesene were exclusively associated with *A. pomi*, whereas linalool, (*E)*-4,8-dimethyl-1,3,7-nonatriene were exclusively associated with *D. plantaginea*. PTR-ToF–MS additionally detected acetic acid (AA) and 2-phenylethanol (PET) in the blends of both trees attacked by aphid species. In the wind tunnel, the aphid predator, *Chrysoperla carnea* (Stephens), responded strongly to a blend of AA and PET, much stronger than to AA or PET alone. The addition of common and species-specific HIPVs did not increase the response to the binary blend of AA and PET. In our setup, two host-associated volatiles AA + PET appeared sufficient in the attraction of *C. carnea.* Our results also show the importance of combining complementary methods to decipher the odor profile associated with plants under pest attack and identify behaviourally active components for predators.

## Introduction

Plants and insects have coexisted on the planet for more than 400 million years. Whereas some of the established interactions are mutualistic, such as pollination, a large number of relationships are antagonistic, such as herbivory (Fürstenberg-Hägg et al. 2013). Herbivory may trigger plant responses that can function both as direct and indirect defenses. Whereas the direct ecological effects of plant response that affect the performance of the attacker and/or the susceptibility of the host (Kessler and Baldwin 2002), the indirect ecological effects of plant response function through the attraction of natural enemies, such as predators and parasitoids. Volatiles released under herbivore attack are termed herbivore-induced plant volatiles (HIPVs) and can play an important role in multi-trophic interactions (Bruce 2014; Dicke and Loon 2000). Because natural enemies of pests are often attracted to plants emitting HIPVs, such *de-novo* released compounds can potentially function as synomones, *i.e.* allelochemicals conveying an advantage to both the emitter and the receiver (Bruinsma & Dicke 2008; Dicke and Baldwin 2010; Gershenzon 2007; Whitfield 2001). Different families of parasitoids and predators, including parasitic wasps (Hymenoptera), hoverflies (Diptera: Syrphidae), predatory bugs (Heteroptera), ladybirds (Coleoptera: Coccinellidae), predatory mites (Mesostigmata), and green lacewings (Neuroptera: Chrysopidae) are attracted to HIPVs in the field (Turlings and Erb 2018).

Natural enemies are fine-tuned to volatiles emitted by plants under attack and navigate upwind in such plumes to find prey (Fatouros et al. 2012). Although volatile signatures associated with chewing herbivores have commonly been shown to be species-specific (Turlings et al. 2000), such response specificity has been studied to a lesser extent with piercing-sucking herbivores, specifically aphids. One of the few examples of aphid induced-specificity was found in the California-native shrub *Baccharis salicifolia*, were two aphid species, one generalist and one specialist induced the release of different quantities of β-myrcene, limonene, and methyl salicylate and unknown. In addition, the researchers found that plant-plant communication following aphid damage was specific to aphid identity (Moreira et al. 2018).

Natural enemies appear to be differentially sensitive to specific HIPVs associated with aphids. For instance, in herbaceous plants like soybean, methyl salicylate was attractive to the predatory lady beetle, *Coccinella septempunctata* (L.), whereas 2-phenylethanol (PET) was more attractive to the lacewing, *Chrysoperla carnea* (Stephens), and syrphid flies (Zhu and Park 2005). On the contrary, the Asian lady beetle, *Harmonia axyridis* (Pallas), showed no preference for any of these compounds (Zhu and Park 2005). HIPVs released from aphid-infested perennial crops are less investigated. Staudt et al. (2010) studied the volatiles induced by *Myzus persicae* (Sulzer) in different peach cultivars and showed quantitative differences in VOCs emissions among peach genotypes, with methyl-salicylate, (*E*)-β-farnesene, (*Z,E*)-α-farnesene, (*E,E*)-α-farnesene (*E*)-β-ocimene and (*E)*-4,8-dimethyl-1,3,7-nonatriene (DMNT) as main released compounds.

Identification of plant volatiles during herbivory is usually carried out via gas-chromatography coupled to mass-spectrometry. However, this methodology does not provide temporal resolution of the HIPV emission and has a low resolution for low molecular weight molecules (Matich et al. 1996). A more sensitive and accurate method to monitor volatiles is proton transfer reaction – time of flight – mass spectrometry (PTR-ToF–MS). To date, only a few studies have investigated the emission of VOCs from herbivore-damaged, woody plants by using PTR-ToF–MS (Giacomuzzi et al. 2016; Peñuelas et al. 2005; Schaub et al. 2010).

In our study, we investigated whether the variation in HIPVs emissions is driven by functional specificity in plant responses to different aphid species. We tested this prediction in apple (*Malus domestica* (Borkhausen) and two aphid species, i.e. the rosy apple aphid (RAA), *Dysaphis plantaginea* (Passerini), and the green apple aphid (GAA), *Aphis pomi* (de Geer). We employed closed-loop stripping analysis (CLSA) followed by gas-chromatography mass-spectrometry (GC–MS) complemented by PTR-ToF–MS to both identify and follow the temporal dynamic of HIPVs induced by the two different aphid species. In addition, wind tunnel bioassays were carried out to investigate the effect of common and species-specific HIPVs on the attraction of the aphid predator *C. carnea*.

## Materials and Methods

*Plant VOC Sampling and Characterization by CLSA-GC–MS—*Volatile compounds were collected from one branch of single apple trees considering three different treatments: RAA-infested trees, GAA-infested trees, and uninfested trees (control). The experiment was conducted in an untreated orchard at the Research Center of Laimburg (Vadena, Italy). A CLSA with a 1.5 mg activated charcoal as adsorbent was used (Brechbühler AG, Schlieren, Switzerland). The trap was fitted to a graphite 12 V vacuum pump (Fürgut, Tannheim, Germany) with Teflon tubes. An apple branch from 8-years old trees (cv Gala grafted on M9 rootstock) infested with an RAA or GAA colony or uninfested was confined into a closed plastic cooking bag (“VOC-bag” 25 × 38 cm, Cuki® oven bag, Cofresco, Volpiano, Italy). Air at 1 l/min was pumped from the bag through the adsorbent trap. For each treatment volatiles were collected from 6 independent trees and lasted 3 h, from 16:00 to 19:00.

The collected samples were eluted from the adsorbent traps with 100 μl GC grade dichloromethane (Sigma-Aldrich, Milan, Italy) and stored at -80 °C prior to GC–MS analysis. VOC samples were analyzed with a GC (7890A) coupled to an MS (5975C Network) (Agilent Technologies, Santa Clara, USA). Two microliters of each sample were injected into the GC port in splitless mode. The GC was equipped with a non-polar HP-5MS column (30 m × 0.25 mm ID, 0.25 μm film thickness, Agilent Technologies). Helium was used as carrier gas at a flow rate of 1.2 ml/min and a velocity of 39.92 cm/s. The mass spectrometric detector was operated in the scan mode (m/z 35 – 400 amu). Data acquisition and analysis were carried out using ChemStation software (Agilent Technologies) and the compounds were identified by comparing their mass spectra with those in the databases NIST 14 (Gaithersburg, MD, USA) and Wiley 10 N (Wiley, Hoboken, NJ, USA). The Kovats retention indices (RI) of the identified VOCs (Van den Dool and Kratz 1963) were calculated using a commercially available mixture of n-alkane standards (nC9-nC20, Sigma-Aldrich), and the obtained RI values were compared with the reference LRI values present in the NIST Chemistry WebBook (Linstrom and Mallard 2018). Moreover, we confirmed the identity of each compound (except β-bourbonene which was commercially unavailable) by comparing the mass spectra and retention times with those of purchased authentic standard compounds.

*PTR-ToF–MS Measurements—*PTR-ToF–MS was used to monitor the VOC emission from a set of apple plants. Two-year-old overwintering potted plants (cv Gala grafted on M9 rootstock) were obtained from a local nursery (Malleier, Lana, Italy). Apple plants were subjected to three independent treatments: RAA infestation, GAA infestation, and undamaged plants as a control treatment. For each treatment volatiles were collected from 5 independent trees. Before the experiments, all plants were grown for 60 d in a growth chamber under a 16-h photoperiod with an L:D temperature regime of 24.0 and 23.0 °C, 60 ± 10% relative humidity, and ca. 90 μmol m^−2^ s^−1^ light intensity. Two days prior to the experiments, the plants were infested with either RAA or GAA by using clip cages (Porcel et al. 2018). Each plant (replicate) was equipped with six clip cages, with six viviparous females of a single aphid species in each clip. Following colony establishment, plants were moved into a climate cabinet (Climacell 707, BMT Medical Technology, Brno, Czech Republic) interfaced with the PTR-ToF–MS via polyether ether ketone (PEEK) capillary tubes (ca. 1.5 m length × 1.01 mm ID, temperature: 110 °C, flow: 40 sccm). The climate cabinet was set with the same parameters as the growth chamber, but with ca. 60 μmol m^−2^ s^−1^ light intensity. Each plant was enclosed within a PFA bag and three capillary tubes were attached to each shoot to be monitored. The three tubes included the following: a perfluoroalkoxy (PFA) tube providing a constant flow of humidified air to the VOC-bag, a second PFA tube removing the overflow air, and a PEEK capillary tube sampling the VOC bag air into the PTR-ToF–MS. As a negative control, volatiles were monitored in parallel on an empty VOC-bag connected to the PTR-ToF–MS with the same tubing system described above. For the three treatments, the experiments were run for 3 days on five plant replicates plus the empty bag.

During the recordings, an automated inlet switching system allowed the PTR-ToF–MS to cycle between the VOC-bags every 2 min, so that the air in each sample could be cyclically analyzed. A gas calibration unit (GCU) instrument (Ionicon Analytik, Innsbruck, Austria) continuously provided each VOC-bag with 5 l/h of humidified (50% relative humidity) zero air. The PTR-ToF–MS (mod. 8000, Ionicon Analytik, Innsbruck, Austria equipped with a time-of-flight detector from Tofwerk AG, Thun, Switzerland) was set to operate in H3O + mode.

*Wind-tunnel bioassay—C. carnea* was purchased as first instar larvae from Biobasiq Sverige AB (Laholm, Sweden). Larvae were reared singly on an artificial diet of *Ephestia kuehniella* (Zeller) eggs. The climate chamber was set at 23 ± 2 °C, 60% RH, and 16:8 h light: dark photoperiod until pupation. Upon emergence, each adult was sexed and kept individually in a vial and provided with water. Behavioural tests were performed in a laboratory wind tunnel with a flight section of 170 × 88 × 70 cm. Humidified air (60% RH, 26 °C) was pushed through custom-made rechargeable cylindrical carbon filters and then into the flight section by a centrifugal fan at a speed of 25 cm/s (Tasin et al. 2012). The central portion of the exhausted airflow with the highest level of semiochemicals was aspirated through the building air system and discarded. A second centrifugal fan pushed the remaining air through the upwind end of the tunnel. The tunnel was illuminated from above by a set of twilight lights with an intensity of 900 lx. The room was kept at 23 ± 2 °C and 40–60% RH. Olfactory stimuli were released from the upwind end of the wind tunnel. Compounds were loaded on a cotton wick as neat synthetics at 1 µl (pure compounds) inside an Eppendorf vial (1.5 ml) with a 2 mm hole in the lid. Paraffin oil (100 µl) was added to the cotton wick. Cardboard holding a vial was hung on a holder at the upwind end of the wind-tunnel. Thirty individuals (15 males and 15 females) were tested for each treatment. In addition, we also tested an empty control treatment (negative control) that consisted of only paraffin. The insects were placed in the wind tunnel room 60 min prior to the experiment to allow acclimation. After the onset of the photophase, batches of two 2–3 day-old lacewings were transferred into a glass tube, which was then placed on a holder at the downwind end of the wind tunnel. The lid facing upwind was then removed to allow the exposition to the odor stimulus. The following behavioral steps were scored for each tested individual; take off (150 cm downwind from the dour), upwind flight at 120, 80 (halfway), and 15 cm (approaching the source) from the odor; landing at the source.

Adult lacewings were exposed to single compounds and blends. Based on the behavioral responses to single compounds, we designed a two-component blend composed of PET and acetic acid (AA) (Sigma-Aldrich, Milan, Italy). After dose–response tests of PET/AA (1, 10 100 µl of each compound), ternary blends were tested by adding a 1 µl of a single additional compound to PET/AA (1 µl). The third compound was selected based on the CLSA-GC–MS results complemented by PTR-ToF–MS data and included benzaldehyde, (*E*)-β-caryophyllene, decanal, ( ±)-linalool (linalool), nonanal, methyl salicylate, (*Z*)-3-hexenyl butyrate, (*Z*)-3-hexenyl-2-methy-butanoate, (*Z*)-3-hexenyl acetate, (*Z*)-3-hexenyl benzoate, p-cymene, DMNT and geranyl acetone (Sigma-Aldrich). β-Bourbonene and (*E*)-β-farnesene were not tested, as the first was not commercially available and of the second there was not enough for behavioural tests.

*Statistical Analyses—*Version 3.5.3 of R was used for statistical analyses and visualization (R Core Team 2013). Kruskal–Wallis rank sum test followed by Dunn's post hoc test was used for analyzing the GC–MS and PTR-ToF–MS output. For the GC–MS data, we compared the peak area of each compound between healthy plants and plants infested with each aphid species. The number of lacewings exhibiting upwind flight or approaching the source was analyzed by a generalized linear model (GLM) with a binomial distribution, using distance*treatments as factors. To discriminate between treatments, trends in glm models were compared using emtrends from the emmeans package (Lenth 2019).

## Results

**Fig. 1:**
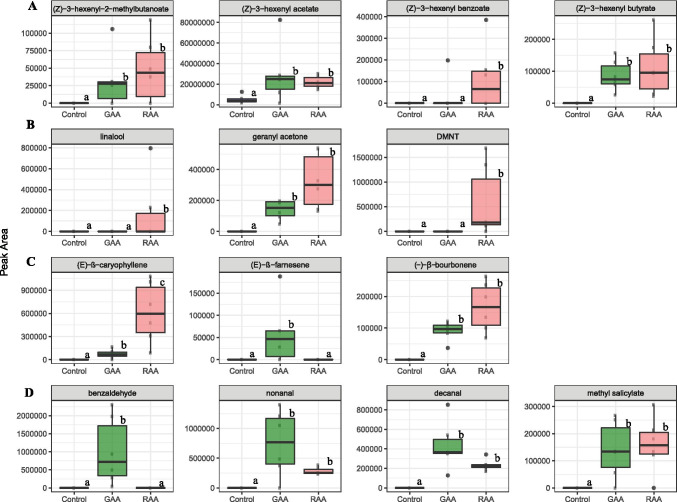
Box plot representation of emission (total ion current of peak area) of herbivore-induced plant volatiles detected by CLSA-GC–MS in the headspace of undamaged apple trees (control) and infested apple tree with green apple aphid (GAA) and rosy apple aphid (RAA). A. green leaf volatiles, B. monoterpenes and DMNT. C. aromatics and aldehydes, D. sesquiterpenes. N = 5. Different letters indicate significant differences among treatments (Kruskal–Wallis rank sum test followed by Dunn's post hoc test, P < 0.05)

**Fig. 2:**
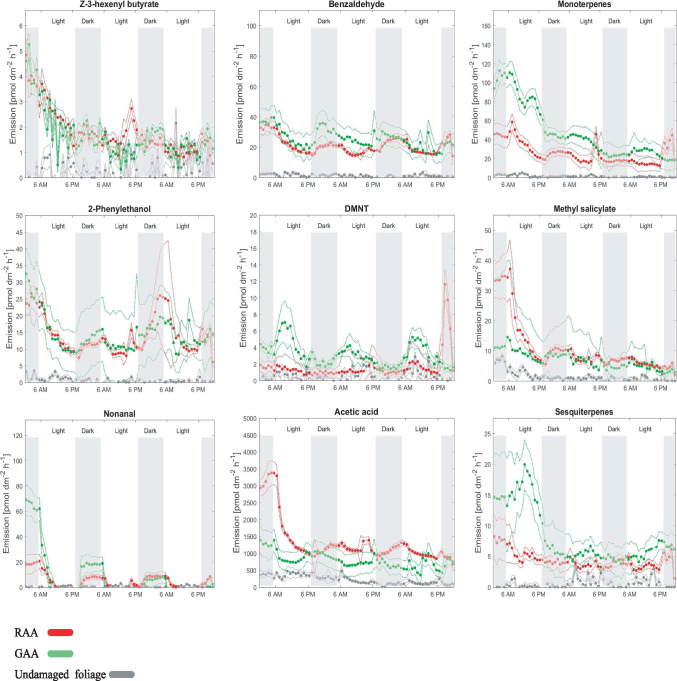
Emission rate (pmol dm^−2^ h^−1^) of volatiles detected by PTR-ToF–MS in the headspace of undamaged apple trees (in red), infested with green apple aphid (in green), rosy apple aphid (in grey) over a period of three days. Plants were infested with aphids 72 h prior to the first measurements. (*Z*)-3-hexenyl butyrate (*m/z* 171.140), benzaldehyde (*m/z* 107.049), monoterpenes (*m/z* 135.119 and 137.131), 2-phenyl ethanol (*m/z* 105.068), DMNT (*m/z* 151.150), methyl salicylate (*m/z* 153.055), nonanal (*m/z* 143.144), acetic acid (*m/z* 61.027), sesquiterpenes (*m/z* 203.167 and m/z 205.196). Graphs show the arithmetic mean of five plant replicates (circles) ± S.E. (dotted lines). White and grey-shaded areas indicate day/night cycle (14 h photoperiod)

*Chemical analysis with CLSA-GC–MS—*The volatile emission from aphid-infested foliage was qualitatively different from that released by healthy plants. The release of some volatiles increased with RAA and GAAinfestations, such as (*Z*)-3-hexenyl acetate (p-values = 0.005 and 0.016, respectively, Fig. 1) and 2-ethyl-1-hexanol, whereas other GLVs were exclusively released upon aphid infestation, sometimes in a species-specific pattern. (*Z*)-3-hexenyl butyrate and (*Z*)-3-hexenyl 2-methyl-butanoate were released upon infestation by both species, whereas (*Z*)-3-hexenyl benzoate was released after RAA infestation and only in one sample after GAA infestation. Terpenoids also displayed a diverse species-selective pattern. Whilst the monoterpene linalool, the homoterpene DMNT, and the sesquiterpene (*E*)-β-caryophyllene were associated with RAA infestation, the sesquiterpene (*E*)-β-farnesene was characteristic of GAA infestation (Fig. 1). In addition, monoterpenoid geranyl acetone and the sesquiterpene β-bourbonene were retrieved from both species. Additional compounds included benzaldehyde, which was released exclusively by GAA attacked plants, and methyl salicylate, decanal, and nonanal, which were associated with infestations of both aphid species (Fig. 1).

*Analysis of on-line volatiles emission by PTR-ToF–MS—*PTR-MS confirmed the emission of the above compounds, but also detected AA and PET that were not detected by GC–MS. Several spectrometric peaks were detected in undamaged as well as aphid infested foliage, including AA, low amounts of GLVs, and sesquiterpenes (Fig. 2), although aphid-infested foliage increased their release. In contrast, methyl salicylate, PET, and benzaldehyde only appeared with aphid infestation.

Several compounds showed diel patterns of release. Monoterpenes, benzaldehyde, DMNT, and nonanal were emitted diurnally, whereas PET was emitted nocturnally (Fig. 2). Monoterpenes, sesquiterpenes, and methyl salicylate emission levels gradually decrease over time, especially from plants with GAA infestation*.* The emission of all the chemical classes from undamaged foliage was low and did not follow a diel pattern. GLVs and AA did not exhibit a distinct diel emission pattern, whether from either intact or aphid-infested foliage. However, the emission of AA (Fig. 2) was found to be higher in aphid infested foliage(p < 0.01 for both aphid species, Kurskall-Wallis test). Volatile emissions of AA and PET did not differ between plants attacked by GAA or RAA.

**Fig. 3:**
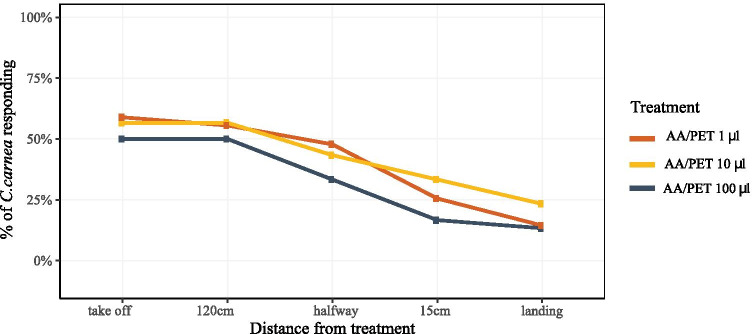
Wind-tunnel dose responses of the lacewing *C. carnea* to the binary blend of 2 phenyl ethanol (PET) and acetic acid (AA). Five parameters taken in the wind tunnel: take off = taking flight, 120 cm = 120 cm flight to lure, halfway = half up-wind to lure, 15 cm = 15 cm flight to lure, landing = landing on the cage containing the lure. No significant differences were found among the three doses tested and between females and males

*Attraction of C. carnea to single compounds, binary and ternary blends –* Wind tunnel bioassay with single compounds showed that only PET elicited upwind flight followed by landing, whereas the response to methyl salicylate elicited oriented upwind flight without landing at the source (Table 1). The combination of AA and PET in a binary blend significantly enhanced the attraction of *C. carnea* (Table 1). The response of adult lacewing to this binary blend was not dose dependent, with the three tested doses eliciting comparable orientation behavior (Fig. 3). The addition of a third compound to the binary blend of AA/PET did not improve the attraction of the tested blends (Table 1). As a tendency, methyl salicylate and linalool did not reduce attraction, while all other tested compounds did (Table 1).

**Table 1:** Wind-tunnel responses of *C. carnea* on synthetic blends of herbivore-induced plant volatiles identified from the headspace of aphid infested apple trees

Behavioral step Blend	Take off	120 cm	Halfway	15 cm	Landing	P-value
Reference blend						
**AA/PET**	59	56	48	26	14	-
Single component						
**PET**	20	20	10	7	7	0.002*
**Methyl salicylate**	20	13	3	3	0	0.002*
**Nonanal**	11	0	0	0	0	< 0.0001*
**Benzaldehyde**	8	0	0	0	0	< 0.0001*
**(*E*)-β*-*caryophyllene**	5	0	0	0	0	< 0.0001*
**Linalool**	5	0	0	0	0	< 0.0001*
**(Z)-3-hexenyl benzoate**	4	0	0	0	0	< 0.0001*
**AA**	0	0	0	0	0	< 0.0001*
**Decanal**	0	0	0	0	0	< 0.0001*
**DMNT**	0	0	0	0	0	< 0.0001*
**Geranyl acetone**	0	0	0	0	0	< 0.0001*
**(*Z*)-3-hexenyl acetate**	0	0	0	0	0	< 0.0001*
**(*Z*)-3-hexenyl butyrate**	0	0	0	0	0	< 0.0001*
**(*Z*)-3-hexenyl-2-methyl-butanoate**	0	0	0	0	0	< 0.0001*
Ternary blend						
**AA/PET/methyl salicylate**	70	67	53	33	20	0.99
**AA/PET/linalool**	57	53	27	17	17	1
**AA/PET/(*E*)-β*-*caryophyllene**	42	31	27	8	8	0.98
**AA/PET/geranyl acetone**	60	50	30	17	7	0.99
**AA/PET/(*Z*)-3-hexenyl butyrate**	83	63	25	13	4	0.12
**AA/PET/(*Z*)-3-hexenyl benzoate**	80	60	30	13	3	0.14
**AA/PET/nonanal**	50	38	23	12	4	1
**AA/PET/(*Z*)-3-hexenyl-2-methyl- butanoate**	59	32	14	5	0	0.99
**AA/PET/decanal**	50	32	14	4	0	1
**AA/PET/(*Z*)-3-hexenyl acetate**	80	63	23	3	0	0.20
**AA/PET /benzaldehyde**	57	37	17	3	0	0.95
**AA/PET/DMNT**	53	27	10	0	0	1

Behavioural responses of *C. carnea* (N = 30) represented as % of individuals performing the behavior. Reference blend of AA/PET was loaded at 1 µl for each compound. Three component blends consisting of AA/PET with an added HIPV was also loaded at 1 µl for each compound. Generalized linear model (GLM) with a binomial distribution was used to analyze the number of lacewings exhibiting upwind flight or approaching the source. The P-values are for the pair comparison test between AA/PET and each single and ternary blends. Five behavioral steps were taken in the wind tunnel: take off = taking flight, 120 cm = 120 cm flight to lure, halfway = half up-wind to lure, 15 cm = 15 cm flight to lure, landing = landing on the cage containing the lure. An asterisk shows a significant difference between the reference bland of AA/PET and each of the other treatments in all the behavioral steps (GLM, p < 0.05). No statistical differences were found between females and males

## Discussion

In this study, the volatiles released by apple foliage infested by RAA or GAA were characterized. The volatile profile of induced apple leaves was affected by the inducing aphid species. The combination of CLSA-GC–MS and PTR-ToF–MS methods with a behavioral bioassay in the wind tunnel, allowed for the identification of behaviorally active components capable of eliciting upwind flight and landing of the aphid predator *C. carnea*.

*Aphid-induced volatiles from apple foliage and other plants—*Interactions between herbivores and plants are complex leading to diverse HIPV blends that differ depending on the host plant and herbivore species (Dicke and Loon 2000). This is also true for aphid-plant interactions. For instance, volatiles associated with pea aphid infestations in alfalfa, partially overlap those reported here, including (*Z*)-3-hexenyl acetate, nonanal, PET, methyl salicylate (*E*)-β-farnesene, and (*E*)-β-caryophyllene (Zhu et al. 2005), whereas pea aphid in broad bean is associated with a rather different volatile profile of linalool and (*E*)-β-farnesene (Du et al. 1998). This again partially overlapped with volatiles associated with *M. persicae* on peach, which comprised of methyl salicylate, (*E*)-β-farnesene, (*Z*, *E*)-α-farnesene, (*E*,*E*)-α-farnesene, DMNT, (*E*)-β-ocimene and (*E*)-nerolidol (Staudt et al. (2010), but even volatile profiles may differ when using the same aphid and host plant species. In apple trees infested with RAA Van Tol et al. (2009) described both similar ((*Z*)-3-hexenyl butyrate, (*Z*)-3-hexenyl acetate, geranyl acetone, methyl salicylate, DMNT and (*E*)-β-caryophyllene), and additional compounds (e.g. 3-carene, α-terpinene, (*E*)-2-hexenyl butyrate, (*E,E*)-α-farnesene) in comparison with our study, underlining that apple varieties and/or volatile analysis methodology can contribute to qualitative differences in reported volatile profiles. In our study, RAA-infested plants were not found to emit (*E*)-β-farnesene, although RAA is reported to directly emit (*E*)-β-farnesene as an alarm pheromone (Francis et al. 2005). On the contrary, GAA-infested plants were found to emit (*E*)-β-farnesene. It is not clear if this compound was released by the plant or produced by GAA as an alarm pheromone. In fact, (*E*)-β-farnesene has not been described as an alarm pheromone for GAA, several species of the same genus (*Aphis fabae* Scop., *Aphis idaei* v.d.Goot, *Aphis sambuci* L., *Aphis urticata* L.) were found to release (*E*)-β-farnesene as an alarm pheromone (Francis et al. 2005). However, *E*)-β-farnesene could also be an induced volatile synthesize from plants as it was recently found that the sesquiterpene synthase OsTPS18 can produce (*E*)-β-farnesene and (*E*)-nerolidol in rice (Kiryu et al. 2018) Moreover, (*E*)-β-farnesene was recently found to be a caterpillar-induced HIPV in infested maize plants (De Lange et al. 2020).

Specifics in aphid-host associations may thus underlie qualitative and quantitative differences and commonalities in volatile profiles. Whether these are functionally significant in indirect defenses, such as the attraction of generalist or specialist parasitoids remains to be investigated. Of interest is that the compounds that appeared critical in attracting *C. carnea* in this study, PET, was identified previously from apple under herbivore attack by larvae of the tortricid moth, *Pandemis heparana* (Denis & Schiffermüller). This was subsequently found to be attractive to *P. heparana* and *P. cerasana* (Hübner) when combined with acetic acid (Giacomuzzi et al. 2016, 2017a), especially when further augmented with the non-induced pome fruit compound ethyl-(2,4)-decadienoate (Larsson Herrera et al. 2020a). Whereas *C. carnea* was previously found attracted to a blend of AA, MS, and phenylacetaldehyde (Pålsson et al. (2019), our study showed for the first time the wind-tunnel attraction of *C. carnea* to a blend of AA and PET. To the best of our knowledge, no studies are available on the behavioral responses *C. carnea* to aphid-associated volatiles from apple trees.

*Combination of analytical methods provides a more complete overview of volatiles released—*Here we demonstrate the value of combining different analytical methods to identify and compare the time dynamics of aphid associated volatiles from apple trees***.*** The PTR-ToF–MS provided high-resolution data on the emissions patterns of VOCs belonging to different chemical classes during aphid feeding, allowing for an on-line monitoring with high temporal resolution. With its very low detection limits (Lindinger et al. 1998) and its mass detection range (20–400 amu (a.m.u.), it permitted detection of compounds that were not be readily detected by solvent-based analytical methods (Peñuelas et al. 2005; Von Dahl et al. 2006). Through PTR-ToF–MS we detected two additional compounds released by infested apple trees, i.e. PET and AA. It is difficult to detect AA with GC–MS methods because the mass is lower than that of the solvent used for the elution of the charcoal filters on a nonpolar column, such as HP5-MS used in our study. On the other hand, acids can interact with polar columns, such as DB-WAX, making the detection/separation of AA troublesome. Few studies investigated the volatile emission from apple trees upon herbivory with PTR-ToF–MS. The majority focused on caterpillars. Giacomuzzi et al. (2016) found that the moth *P. heparana* induced the release of several compounds that were also found in our study, such as methyl salicylate, linalool, (*Z*)-3-hexenyl acetate, Z3hbe, (*E*)-β-caryophyllene, and DMNT.

The PTR-ToF–MS offers detailed time resolution on the emission of the volatiles, something that is difficult to obtain using GC–MS. For instance, Giacomuzzi et al. (2017b) found using PTR-ToF–MS that apple trees have diurnal peaks in the emission of terpenes. In our study, such diel patterns in the release were also observed, with diurnal peak emissions for monoterpenes, benzaldehyde, DMNT, and nonanal. PTR-ToF–MS also showed a delay in the emission of terpenes after *P. heparana* infestation, consistent with the premise that plants need time to mount an induced response and systemically activate the biosynthetic pathways of these secondary metabolites (Dudareva et al. 2013). In our study, infested and non-infested plants differed in volatile emission from the first day of monitoring, likely since aphid colonies were already established on the apple trees for at least 3 days.

### The Origin of ‘Induced’ Volatiles

In this study, the composition of volatile blends was aphid species dependent. Such blends carry a signature of the underlying biochemical processes, whether of plant or microbial origin. Whereas aphids can induce indirect defenses in plants, they also secrete a large amount of honeydew, which consists of sugars and amino acids and provides a rich growth medium for microbes (Leroy 2011a). Volatiles associated with aphid infestation can thus be of plant and/or microbial origin. As the composition some of the sugars and amino acids are also synthesized by aphid species (Shaaban et al. 2020) and associated endosymbiotic bacteria associated with aphids (Febvay et al. 1999), the composition of volatile blends associated with aphid infestation can be species dependent.

Determining which compounds are of plant or of microbial origin may be difficult. For example, PET is a common compound synthesized by plants from phenylalanine (Bruce et al. 2005) and is found in a wide array of flowers and fruits, including apple (Buchbauer et al. 1993; Knudsen et al. 1993; Omata et al. 1990). However, PET is also found in the honeydew of the pea aphid *Acyrthosiphon pisum* (Peach et al. 2019). Indicative of microbial activity, Leroy (2011b) found that the bacteria *Staphylococcus sciuri* isolated from aphid honeydew could produce PET, whose release could be of either plant and/or microbial origin. Another important compound in our study is the well-known fermentation volatile AA (De Roos and De Vuyst 2018). Detecting AA in the headspace of plants could indicate the presence of microbial activity. Orientation to PET and AA may have originated from the need for protein for opportunistic feeders such as lacewings, as it indicates active fermentation, as well as protein sources in the form of microbes and aphids.

### Behavioral Importance of PET & AA and Other Compounds

Both PET and AA have been extensively studied previously in insect species. The combination of these compounds attracted *Drosophila melanogaster* (Meigen), *Choristoneura rosaceana* (Harris), *P. heparana* (Becher et al. 2010; Giacomuzzi et al. 2016; Larsson Herrera et al. 2020a; Knight et al. 2017; Zhu et al. 2003). Larsson Herrera et al. (2020b) found that this combination attracts several species, including *L. botrana* and *C. carnea*, and the specificity of this binary blend depended on the release rates of these two compounds. In particular, a low amount of PET in the blend reduced the catches of *C. carnea*. Lucchi et al. (2017) and Jones (2016) observed in apple, pear, walnut, and grapevine orchards, that a combination of methyl salicylate + AA + PET was able to attract *C. carnea*. This fits with our behavioral data. A blend of the above three volatiles was the only combination that induced a slightly better orientation of *C. carnea* compared to AA + PET (Table 1). Other studies in field crops and fruit orchards, showed that a combination of methyl salicylate + AA + phenylacetaldehyde can attract *C. carnea (*Koczor et al. 2019; Pålsson et al. 2019*)*. Methyl salicylate, a benzenoid derivative from the salicylic acid pathway, is among the most commonly emitted HIPV and it has been reported to be involved in the recruitment of a number of natural enemies (Salamanca et al. 2015; Tóth et al. 2009).

Whereas thus PET and AA induced upwind orientation and landing for *C. carnea*, additional compounds associated with aphid infestations did not further enhance attraction. The ecological significance of these additional volatiles in lacewing attraction thus remains uncertain, although it was shown that benzaldehyde alone can attract *Chrysoperla plorabunda* in the field (Fitch) (Jones et al. 2011). It may be that in our study the combination of AA and PET, presented at high concentrations, blurred possible additive or synergistic effects of the other compounds. Further tests with more complete blends of aphid associated volatiles, perhaps in release rates that reflect natural concentrations, and possibly in combination with apple plant volatiles are warranted. At the same time, a generalist predator such as C. carnea may respond to the general part of the blend, such as AA and PET, whereas more specialized natural enemies exploit specific differences in the induced blends (McCormick et al. 2012).

Only a few studies have investigated the orientation and landing for *Chrysoperla* spp. in the wind tunnel. Han and Chen (2002) showed that volatiles from shoots of tea plants, *Camellia sinensis* (L.), damaged by *Toxoptera aurantii* were more attractive to *Chrysoperla sinica* than those released by undamaged plants. Moreover, benzaldehyde elicited an increased upwind flight in *C. sinica* compared with other volatiles. In contrast, in our study benzaldehyde was singly not behaviorally active at the tested dose.

It remains unknown whether the volatiles identified in the present study induce feeding, oviposition, or a combination of these. In a study of Ballal and Singh (1999), *C. carnea* laid a significantly higher number of eggs on sunflower, compared to cotton. The underlying attraction to sunflower could be ascribed to PET, which is released from this species (Zhu et al. 2005). The field experiment of Pålsson et al. (2019), showed a higher number of *C. carnea* eggs in the vicinity of baits emitting methyl salicylate, AA, and phenylacetaldehyde. Although not tested in our study, oviposition may thus be similarly triggered by the blend of AA and PET, in which PET might act as a replacement for PAA and MS.

## Conclusion

We demonstrated here that the composition of the headspace volatiles of infested apple trees depended on the aphid species. Our study highlights the importance of combining methods to map the dynamics and detect a full range of volatiles released upon aphid infestation. We found that a synthetic blend of two compounds, AA and PET, induced by both aphid species, was attractive to *C. carnea*. Interestingly, no compound added to the two-component blend significantly increased attraction. Future studies are needed to dissect the role of indirect defense, honeydew, and/or microorganisms function in attracting natural enemies. These future studies should consider how blend ratio, composition, and release rates affect attractiveness to natural enemies and non-target species.
